# Immediate Molar Implant Placement With Planned Immediate Restorative Management: A Retrospective Descriptive Case Series

**DOI:** 10.7759/cureus.119055

**Published:** 2026-11-03

**Authors:** Nicholas Fahey, Gregori M Kurtzman

**Affiliations:** 1 Dentistry, Private Practice, London, GBR; 2 Dentistry and Implantology, Private Practice, Silver Spring, USA

**Keywords:** digital implant planning, immediate implant placement, immediate provisionalization, molar extraction socket, retrospective case series

## Abstract

Background: Immediate molar implant placement requires management of complex socket anatomy, infection, residual bone, and posterior loading. This retrospective descriptive case series evaluates the recorded clinical use of a restoratively driven protocol with planned immediate restorative management.

Materials and methods: This retrospective descriptive series included 46 patients with 46 molar sites treated over the preceding five years, comprising 23 maxillary and 23 mandibular sites in private practice. The series comprised 46 patients with one listed treatment site per patient, treated during the five years preceding this retrospective review. Eligible cases involved failing molars planned for immediate implant placement; the sole exclusion criterion was an extraction site too large to permit immediate placement. Nicholas Fahey performed all procedures and managed or reviewed the available follow-up records. Patients were reviewed at two and eight weeks, and the restorative phase was initiated at 12 weeks. Site-level data were summarized descriptively.

Results: Pre-existing infection was recorded in 37 of 45 sites with documented status (82.2%); one status was missing. Forty-five entries recorded insertion torque of 25-80 N·cm. Provisional materials were zirconia in 24 sites, polymethyl methacrylate in 18, and composite in three. The reported complications were postoperative pain in maxillary case 12, a loose custom healing abutment in maxillary case 15 that was tightened at the initial postoperative visit, and slight recession at the custom healing abutment in mandibular case 15. Mandibular case 20 lacked primary stability, and placement of the custom healing abutment was delayed. No additional complications were documented in the available records. Illustrated follow-up extended to 53 weeks in the maxillary case and two years in the mandibular case.

Conclusions: This retrospective series describes the clinical application of immediate molar implant placement with planned immediate restorative management in private practice. The reported complications were limited, and the protocol was modified in one site by delaying custom healing abutment placement when primary stability was inadequate. Complete site-level observation periods and standardized endpoint data are needed to estimate implant survival or comparative effectiveness.

## Introduction

Immediate implant placement has moved from a procedure used selectively in the esthetic zone to a treatment option that can also be considered in posterior extraction sites. The attraction is straightforward: if a hopeless tooth can be removed atraumatically, an implant positioned in a prosthetically correct location, and a provisional tooth delivered at the same appointment, the clinician may be able to reduce the number of surgical stages while maintaining the anatomy that already exists.

The molar socket is not simply a larger anterior socket. Its anatomy is more complex. The clinician must manage divergent roots, a broad extraction socket, bone loss, variable interradicular bone, and frequently chronic periapical or furcation infection. In the maxilla, residual bone beneath the sinus may be limited. In the mandible, implant length and apical engagement are constrained by the inferior alveolar canal. These factors explain why staged extraction, socket preservation, healing, implant placement, and later restoration have traditionally been favored.

That staged approach remains appropriate in many patients, but changes to the ridge begin as soon as the tooth is removed. Following extraction, the ridge undergoes horizontal and vertical dimensional change, with substantial remodeling during healing [1,2]. Delaying implant placement may therefore mean reconstructing anatomy that was present at the time the tooth was removed. The concept behind the technique described here is to maintain as much of that anatomy and restorative space as possible rather than regain it later. This was the clinical rationale; no ridge-dimensional preservation outcome was measured in this series.

Published evidence supports immediate implant placement in selected molar sockets. A systematic review and meta-analysis of 1,106 immediately placed molar implants reported favorable weighted one-year survival, although outcomes were influenced by factors including grafting, loading protocol, and buccal bone condition [3]. Large clinical series have also documented favorable outcomes for immediate placement at both maxillary and mandibular molar extraction sites [4,5]. These data do not make every molar socket an immediate placement site; they support careful selection and a disciplined technique.

The primary objective of this retrospective descriptive case series was to describe the recorded clinical workflow for immediate molar implant placement with planned immediate restorative management. Secondary descriptive variables were clinically documented infection, insertion torque, recorded restorative material, soft-tissue grafting, and reported adverse events. Two selected illustrated cases provided procedural examples rather than representative outcome evidence for the full series.

## Materials and methods

Study design and clinical records

This retrospective descriptive case series included maxillary and mandibular molar sites treated in private practice. All cases were treated, verified, and followed by the lead author, Nicholas Fahey. The treating author confirmed that the 46 listed cases represented 46 individual patients, each contributing one molar site, treated over the preceding five years. The exact start and end dates and whether all eligible cases were consecutive were not specified.

Eligibility and case selection

Cases were selected when a failing maxillary or mandibular molar was planned for immediate implant placement. The sole exclusion criterion was an extraction site too large to permit immediate implant placement at the failing molar. No additional study exclusion criteria were applied. The patients presented to the practice with failing molars and were planned for implant replacement. A numerical socket-size cutoff and whether all eligible patients were included consecutively were not specified.

Ethics and consent

All patients received treatment in private practice. Written informed consent for treatment and open-access publication was obtained from all patients. No patient-identifying information was included in the manuscript or accompanying images. Institutional review board review was not required for this retrospective study, as reported by the authors. The authors reported that IRB review was not required because treatment occurred in private practice and the analysis was retrospective. Each patient signed a treatment consent form. No formal committee exemption or supporting policy was supplied for this revision.

Planning and treatment protocol

Cone beam computed tomography (CBCT) imaging and an intraoral scan were obtained before surgery. The restorative endpoint was established first by creating a virtual diagnostic setup of the tooth and soft tissue in the ideal position. Implant planning was then performed around the proposed restoration rather than simply centering the implant within the extraction socket or following empirical guidelines. This approach was particularly important in molar sites, where the center of the socket did not necessarily correspond to the ideal restorative trajectory.

The treatment plan identified where native bone would provide implant stability. Depending on socket anatomy, this included interradicular or septal bone and apical bone beyond the root apices. In the maxilla, the relationship to the sinus floor was evaluated, and internal sinus augmentation was incorporated when indicated. In the mandible, the available vertical dimension above the inferior alveolar canal was evaluated and respected during implant planning.

The clinical sequence established the restorative endpoint before extraction. Treatment included atraumatic extraction, complete socket debridement, verification of the surgical guide, preparation of a restoratively guided osteotomy using a custom surgical guide in every case, implant placement, management of the residual implant-socket gap, and delivery of an anatomically contoured provisional restoration or custom healing abutment when primary stability permitted. Primary stability was assessed at implant placement. In mandibular case 20, custom healing abutment placement was delayed because primary stability was inadequate.

A custom surgical guide was used for every case. Dynamic navigation was mentioned in the illustrated mandibular narrative, but its additional use and system require confirmation. The molar was sectioned so the roots could be removed individually whenever possible. Preservation of the socket walls and interradicular bone was prioritized because the remaining native bone was important for both implant positioning and primary stability. Root removal was directed away from the remaining socket walls rather than using the socket itself as a fulcrum.

Following extraction, granulation tissue and residual pathologic soft tissue were meticulously removed. In the clinical protocol used for these cases, the socket was additionally treated with 1.5% chlorhexidine gel (OraCare Reassure Gel, OraCare, Bridgeport, WV) for approximately three minutes and then irrigated. The published literature on infected sockets had emphasized thorough mechanical debridement, while evidence regarding specific adjunctive chemical decontamination protocols remained less standardized [6-9].

The treating author identified the adjunct as Chlosite Gel (Ghimas, Casalecchio di Reno, Italy), a xanthan-based gel containing 1.5% chlorhexidine, comprising 0.5% chlorhexidine digluconate and 1% chlorhexidine dihydrochloride. The earlier chlorine dioxide designation was incorrect. Its independent contribution was not evaluated in this uncontrolled series.

The surgical guide was then seated, and its stability was verified on the adjacent dentition. Osteotomy preparation was performed according to the digitally planned implant position rather than simply following the center of the extraction socket. Low-speed drilling was used in the treatment protocol, allowing autogenous bone collected from the drill flutes to be retained for grafting. The treating author specified drilling at 800 rpm with saline irrigation. The relationship between this general protocol and the separately described reverse-drilling step was not specified.

The implant was positioned in native bone according to the restorative plan. Primary stability guided the timing of connection of the provisional restoration or custom healing abutment. When primary stability was inadequate in mandibular case 20, the lead author delayed custom healing abutment placement.

The stated criterion for immediate restorative connection was insertion torque of at least 35 N·cm. Mandibular case 20 did not achieve adequate primary stability, and custom healing abutment placement was delayed for 12 weeks before restoration. Its numerical insertion torque and interim closure were not supplied. Maxillary case 15 recorded 25 N·cm and a loose custom healing abutment; this requires clarification as an exception to the stated threshold.

Residual defects between the implant and socket walls were grafted selectively. In the protocol used for these cases, autogenous bone collected during osteotomy preparation was combined with particulate allograft and platelet-derived material. Barrier membrane and/or connective-tissue grafting were added when the buccal plate or soft-tissue volume required additional support. Grafting was used to manage residual defects after acceptable implant positioning and primary stability had been achieved; it did not correct implant malposition or inadequate stability.

Graft materials and adjuncts varied. The maxillary example used OsteoBiol GTO (Tecnoss, Giaveno, Italy), whereas the mandibular example used MinerOss cortical and cancellous chips with autogenous bone and platelet-derived material. The frequency of each graft combination, membrane product, platelet preparation system and centrifugation protocol, and objective allocation criteria were not recorded systematically. Platelet-rich fibrin (PRF) and leukocyte- and platelet-rich fibrin (L-PRF) labels were retained as originally described and were not assumed to indicate identical preparations.

An anatomically contoured CAD/CAM zirconia provisional restoration was connected at implant placement in both illustrated cases. The provisional was contoured to support the extraction socket and peri-implant soft tissues, maintain proximal contact and mesiodistal space, and reproduce the emergence form intended for the definitive restoration. It was adjusted to remain out of functional occlusion during early healing.

These CAD/CAM zirconia crowns were specific to the illustrated cases. The full series also contained PMMA and composite material entries. Nonfunctional provisionalization meant the intended absence of centric and excursive contacts, but site-level documentation of the verification method or compliance was not available.

The provisional remained in place while osseointegration and tissue maturation occurred. At the restorative visit, it was removed, the peri-implant tissues were evaluated, a scan body was placed, and a digital impression was obtained. A screw-retained zirconia definitive restoration was then fabricated and delivered after verification of contacts, emergence profile, radiographic seating, and occlusion. Twelve weeks denoted the planned initiation of the restorative phase rather than a uniform definitive delivery date. Actual scanning and definitive delivery intervals were not available for all sites.

Data extraction and outcomes

The lead author performed all procedures and reviewed the available follow-up records. Site-level data were derived from the clinical tables and included tooth site, implant system and dimensions, infection designation, insertion torque, provisional material, soft-tissue grafting, and complications. Tooth numbers were recorded using the Fédération Dentaire Internationale (FDI) two-digit notation. Infection was defined clinically by the presence of exudate and/or a fistula at the failing molar. Torque values represented insertion torque rather than implant stability quotient measurements. The authors clarified the documented complications and their management after reviewing the draft. Clinically documented infection referred specifically to recorded exudate and/or a fistula. A negative designation did not exclude other endodontic or periapical disease. Complication descriptions included retrospective clarification by the treating author; contemporaneous chart verification was not demonstrated in the supplied materials.

Follow-up assessment

Patients were seen two weeks after implant placement for suture removal and site evaluation and again eight weeks after surgery. The restorative phase was initiated at 12 weeks. All follow-up evaluations were performed by Nicholas Fahey. The illustrated records included clinical photographs, periapical radiographs, and CBCT examinations, with maxillary documentation extending to 53 weeks and mandibular documentation extending to two years. The treating author reported that no patients were lost to follow-up, with maxillary follow-up to 53 weeks and mandibular documentation to two years. Site-specific dates and visit-level completion records were not supplied, so these reported intervals were not converted into a survival analysis or assumed to be identical observation times for every site.

Descriptive analysis

The treatment site was the unit of descriptive analysis. The supplied clinical summary tables contained 46 populated site records. An empty mandibular table row contained no clinical record and was not an additional case. Infection percentages were calculated using sites with recorded infection status. Provisional material counts combined PMMA, LR6 PMMA, and LR7 PMMA as polymethyl methacrylate (PMMA); mandibular case 20 was reported separately because custom healing abutment placement was delayed.

The original "NO PRP" annotation was treated as a procedural note rather than a complication. Complication descriptions were reconciled with the authors' clarification, and no additional complications were documented in the available records. Missing data for other variables were retained as missing. No inferential comparisons were performed. Implant survival and time-dependent complication rates were not calculated because complete per-site observation periods and standardized endpoints were not supplied.

Available-case denominators were used for each reported variable: infection 45/46 sites with one missing status, numeric torque 45/46, and recorded restorative material 45/46. Material counts described materials only, not verified counts of immediate crowns versus healing abutments. No imputation or patient-level inferential analysis was performed.

## Results

Site characteristics and recorded treatment

The series contained 23 maxillary site entries (Table 1) and 23 mandibular site entries (Table 2). Infection was recorded in maxillary sites (n = 18/23; 78.3%) and mandibular sites with known status (n = 19/22; 86.4%); one mandibular status was missing. Across both arches, documented entries recorded infection (n = 37/45; 82.2%). These are site counts and should not be interpreted as the number of infected patients. Recorded materials were zirconia in 24 sites, PMMA in 18, and composite in three; mandibular case 20 had delayed custom healing abutment placement. Exact counts of immediate crowns versus customized healing abutments remained unconfirmed. The treating author reported no significant postoperative pain; the earlier maxillary case 12 pain entry was retained as a recorded symptom, without implying clinically significant pain. Loosening in maxillary case 15 and slight recession in mandibular case 15 remained recorded events. These counts were not standardized complication rates.

**Table 1:** Maxillary immediate molar implant site records. FDI: Fédération Dentaire Internationale; PMMA: polymethyl methacrylate; PL: Progressive-Line; TPC: Tapered Pro Conical. L denotes implant length in mm. Numerical insertion torque replaces undefined low, medium, and high labels. Not recorded denotes missing documentation. None retains the source designation of no graft, and N/A retains the source notation. No additional event documented refers only to the available records and does not confirm an uneventful standardized follow-up interval. Restorative material does not establish component type or timing.

Case	FDI site	Implant and size (mm)	Clinical infection	Insertion torque (N·cm)	Recorded restorative entry	Soft-tissue graft	Recorded event
1	26	CONELOG PL; 5.0 L9	Yes	60	Zirconia	None	No additional event documented
2	16	BioHorizons TPC; 5.2 L10.5	Yes	60	Zirconia	None	No additional event documented
3	26	BioHorizons TPC; 5.2 L9	No	60	Zirconia	Not recorded	No additional event documented
4	26	CONELOG PL; 5.0 L9	Yes	35	PMMA	None	No additional event documented
5	27	CONELOG PL; 5.0 L9	Yes	35	PMMA	Not recorded	No additional event documented
6	17	CONELOG PL; 5.0 L9	Yes	80	PMMA	None	No additional event documented
7	26	BioHorizons TPC; 5.2 L9	Yes	75	Zirconia	None	No additional event documented
8	26	CONELOG PL; 5.0 L9	Yes	75	Zirconia	Connective tissue graft	No additional event documented
9	16	CONELOG PL; 5.0 L7	Yes	80	PMMA	None	No additional event documented
10	17	CONELOG PL; 5.0 L7	Yes	35	Zirconia	None	No additional event documented
11	17	CONELOG PL; 5.0 L11	No	75	Zirconia	None	No additional event documented
12	16	CONELOG PL; 5.0 L9	Yes	70	Zirconia	Connective tissue graft	Postoperative pain
13	26	CONELOG PL; 5.0 L9	Yes	80	PMMA	None	No additional event documented
14	16	CONELOG PL; 5.0 L11	Yes	35	PMMA	None	No additional event documented
15	16	CONELOG PL; 5.0 L9	No	25	Zirconia	None	Loose custom healing abutment; tightened
16	16	CONELOG PL; 5.0 L9	No	80	Composite	None	No additional event documented
17	16	CONELOG PL; 5.0 L7	Yes	35	PMMA	None	No additional event documented
18	26	CONELOG PL; 5.0 L9	Yes	75	PMMA	None	No additional event documented
19	16	CONELOG PL; 5.0 L11	Yes	80	PMMA	None	No additional event documented
20	26	CONELOG PL; 5.0 L9	Yes	80	Zirconia	None	No additional event documented
21	16	CONELOG PL; 5.0 L9	No	35	PMMA	None	No additional event documented
22	26	CONELOG PL; 5.0 L9	Yes	75	PMMA	None	No additional event documented
23	27	CONELOG PL; 5.0 L9	Yes	80	PMMA	None	No additional event documented

**Table 2:** Mandibular immediate molar implant site records. FDI: Fédération Dentaire Internationale; PMMA: polymethyl methacrylate; PL: Progressive-Line; TPC: Tapered Pro Conical. L denotes implant length in mm. Numerical insertion torque replaces undefined low, medium, and high labels. Not recorded denotes missing documentation. None retains the source designation of no graft, and N/A retains the source notation. No additional event documented refers only to the available records and does not confirm an uneventful standardized follow-up interval. Restorative material does not establish component type or timing.

Case	FDI site	Implant and size (mm)	Clinical infection	Insertion torque (N·cm)	Recorded restorative entry	Soft-tissue graft	Recorded event
1	37	CONELOG PL; 5.0 L13	Yes	35	Zirconia	None	No additional event documented
2	36	CONELOG PL; 5.0 L11	Yes	60	PMMA	None	No additional event documented
3	46	CONELOG PL; 5.0 L11	Yes	35	Zirconia	None	No additional event documented
4	36	CONELOG PL; 5.0 L13	Yes	75	Zirconia	None	No additional event documented
5	46	CONELOG PL; 5.0 L13	Yes	75	Zirconia	None	No additional event documented
6	37	CONELOG PL; 5.0 L11	Yes	80	Zirconia	None	No additional event documented
7	38	CONELOG PL; 5.0 L11	Yes	35	Zirconia	None	No additional event documented
8	36	CONELOG PL; 5.0 L11	Yes	80	Zirconia	Connective tissue graft	No additional event documented
9	46	BioHorizons TPC; 5.2 L10.5	Yes	35	PMMA	Connective tissue graft	No additional event documented
10	46	BioHorizons TPC; 5.2 L15	Yes	75	Zirconia	Connective tissue graft	No additional event documented
11	46	BioHorizons TPC; 5.2 L12	Yes	75	PMMA	Connective tissue graft	No additional event documented
12	46	BioHorizons TPC; 5.2 L12	No	80	PMMA	None	No additional event documented
13	36	BioHorizons TPC; 5.2 L12	Yes	80	Zirconia	None	No additional event documented
14	46	BioHorizons TPC; 5.2 L10.5	Yes	75	Zirconia	Connective tissue graft	No additional event documented
15	46	CONELOG PL; 5.0 L11	Yes	75	Zirconia	None	Slight recession at custom healing abutment
16	36	CONELOG PL; 5.0 L11	Yes	80	Zirconia	None	No additional event documented
17	36	CONELOG PL; 5.0 L13	Yes	80	Zirconia	Connective tissue graft	No additional event documented
18	46	CONELOG PL; 5.0 L13	Yes	70	LR6 PMMA	None	No additional event documented
19	47	CONELOG PL; 5.0 L13	Not recorded	70	LR7 PMMA	Not recorded	No additional event documented
20	36	CONELOG PL; 5.0 L9	No	Not obtained	Custom healing abutment placement delayed	N/A	Inadequate primary stability; healing abutment delayed
21	46	CONELOG PL; 5.0 L9	No	60	Zirconia	None	No additional event documented
22	46	CONELOG PL; 5.0 L9	Yes	80	Composite	None	No additional event documented
23	36	CONELOG PL; 5.0 L9	Yes	80	Composite	None	No additional event documented

Illustrated case 1 maxillary first molar

A 59-year-old woman presented with a structurally compromised maxillary first molar after loss of the previously placed crown. Radiographic evaluation demonstrated bone loss and periapical pathology (Figure 1), and clinical examination confirmed the compromised remaining tooth structure (Figure 2A, B). Preoperative CBCT-based virtual planning was performed to establish the restoratively driven implant position and evaluate the available bone and its relationship to the maxillary sinus floor (Figure 3A, B).

**Figure 1:**
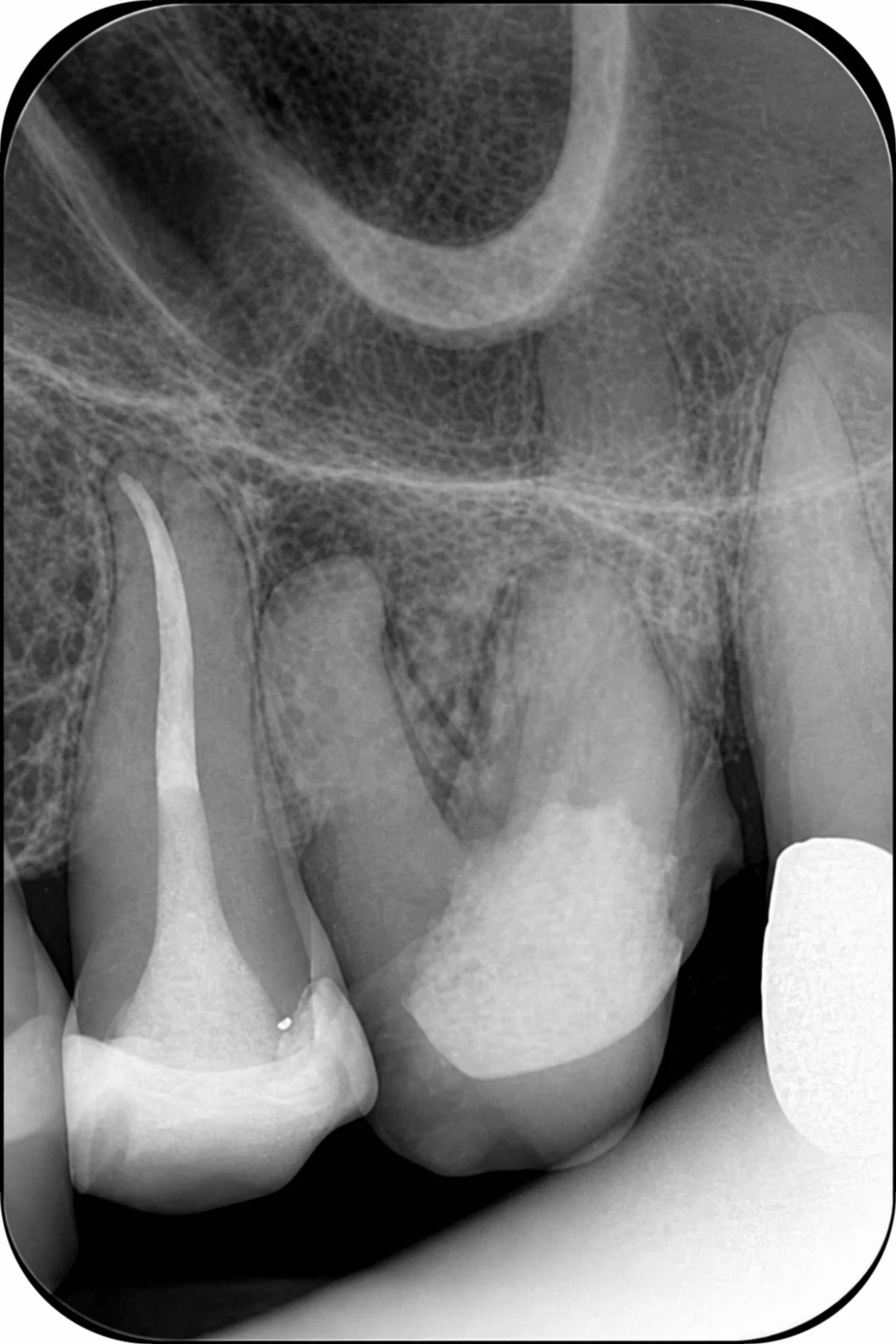
Preoperative periapical radiograph of the compromised maxillary molar, demonstrating the remaining tooth, surrounding bone, and periapical region.

**Figure 2:**
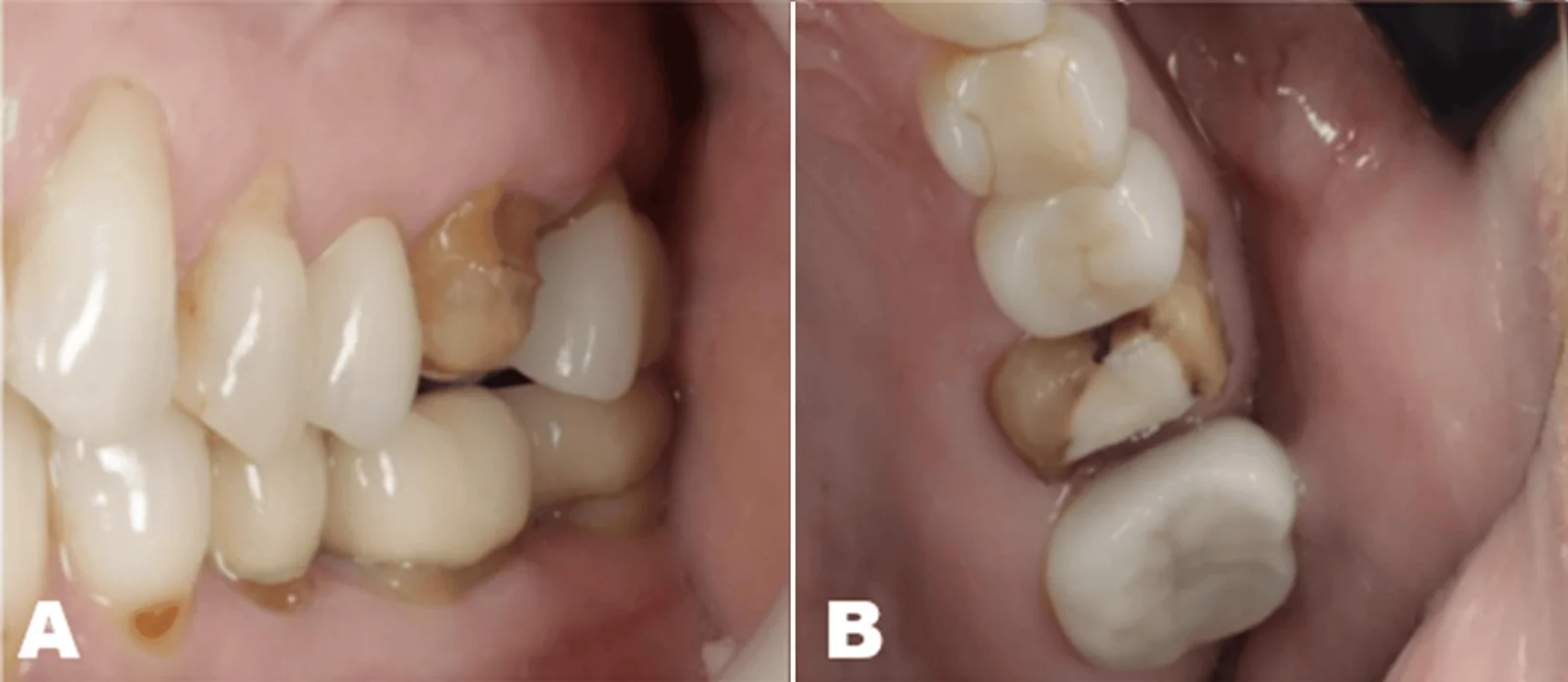
Preoperative clinical views after loss of the maxillary molar crown. (A) Buccal view of the remaining tooth and adjacent dentition. (B) Occlusal view of the compromised molar.

**Figure 3:**
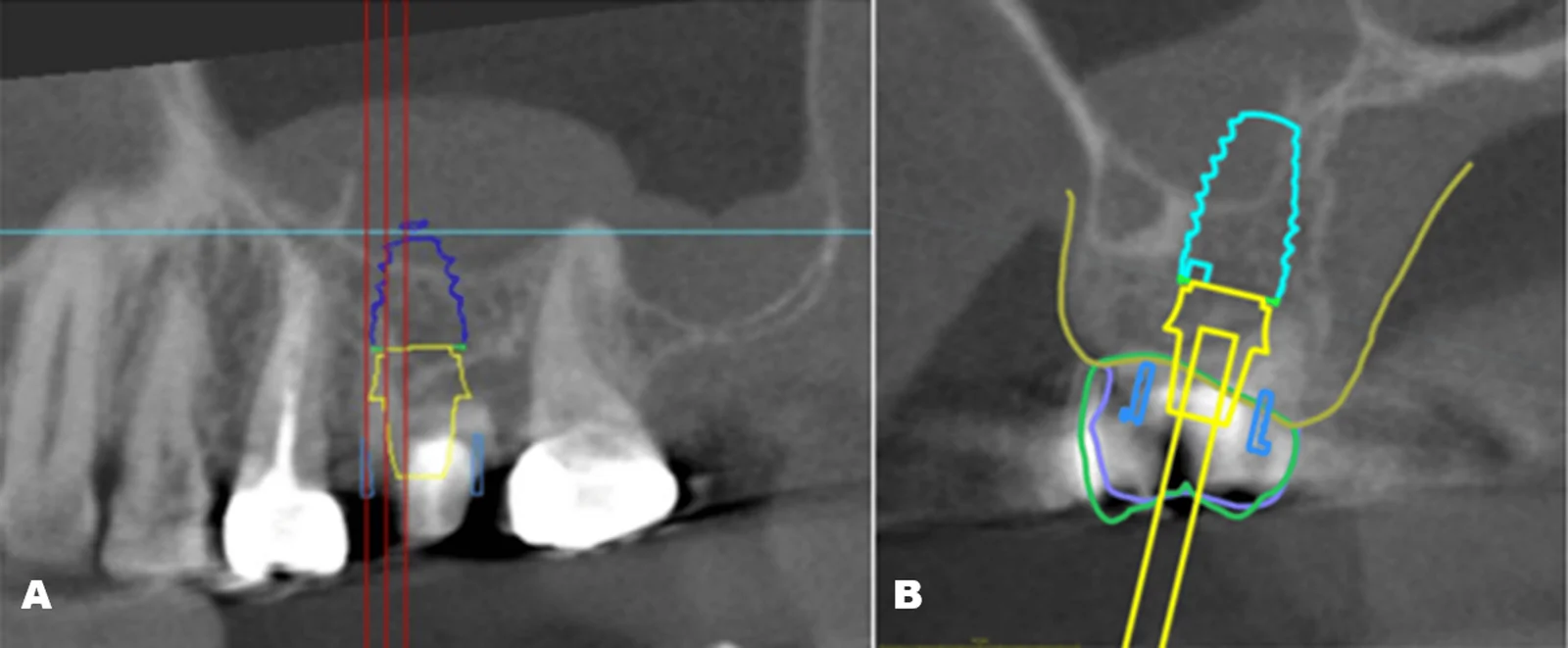
Restoratively driven virtual implant planning in the posterior maxilla. (A) Reconstructed view showing the proposed implant position relative to the adjacent teeth. (B) Cross-sectional view showing the planned implant and its relationship to the sinus floor.

The treating author identified this illustrated patient as maxillary case 4 in Table 1. That row recorded a CONELOG Progressive-Line Implant measuring 5.0 × 9 mm with insertion torque of 35 N·cm, PMMA, and no soft-tissue graft. The illustrated narrative instead reported 74 N·cm, a zirconia provisional, and a tuberosity connective tissue graft. These details require reconciliation against the original record.

Following atraumatic extraction, socket debridement, and disinfection (Figure 4A), the socket was evaluated. A 9-mm CONELOG Progressive-Line implant was placed in the digitally planned position with engagement of native bone and insertion torque not verified against a linked table entry. A crestal sinus lift was performed with osteotomes; no sinus membrane perforation was documented. Autogenous bone was delivered using a short 7-mm form drill in reverse. The residual socket space was grafted with autogenous bone and OsteoBiol GTO (Tecnoss), hydrated with PRF (Figure 4B). A connective tissue graft from the tuberosity was added, and an anatomically contoured zirconia provisional restoration was immediately connected (Figure 4C).

**Figure 4:**
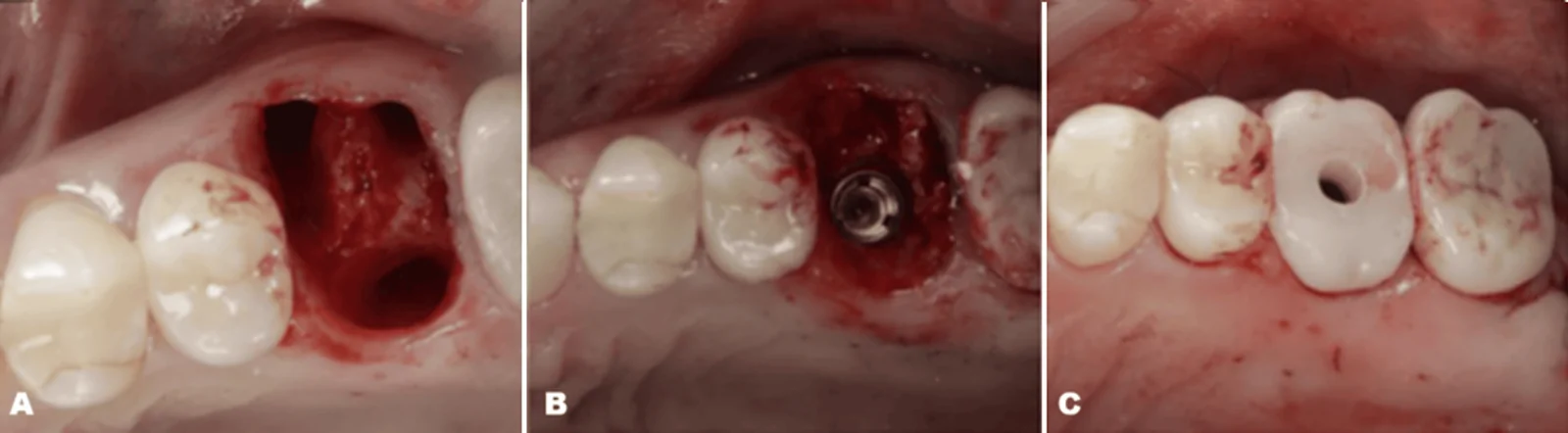
Maxillary surgical and immediate restorative sequence. (A) Extraction socket after removal of the failing molar and debridement. (B) Implant positioned within the socket before provisional restoration. (C) Occlusal view after connection of the anatomically contoured provisional restoration.

Residual subantral height, intended elevation, formal membrane testing, reverse-drilling details, PRF processing, and the specific indication for tuberosity grafting were not supplied. No membrane perforation was documented; this did not establish that a formal integrity test was performed. A placement periapical radiograph documented the implant and immediate provisional restoration (Figure 5). Radiographs at definitive restoration and at 28 weeks documented the radiographic appearance of crestal bone (Figure 6A, B). Follow-up CBCT documented the implant and augmented posterior maxillary anatomy at 53 weeks (Figure 7A, B). At the same follow-up, the peri-implant gingiva showed no visible inflammation (Figure 8A, B).

**Figure 5:**
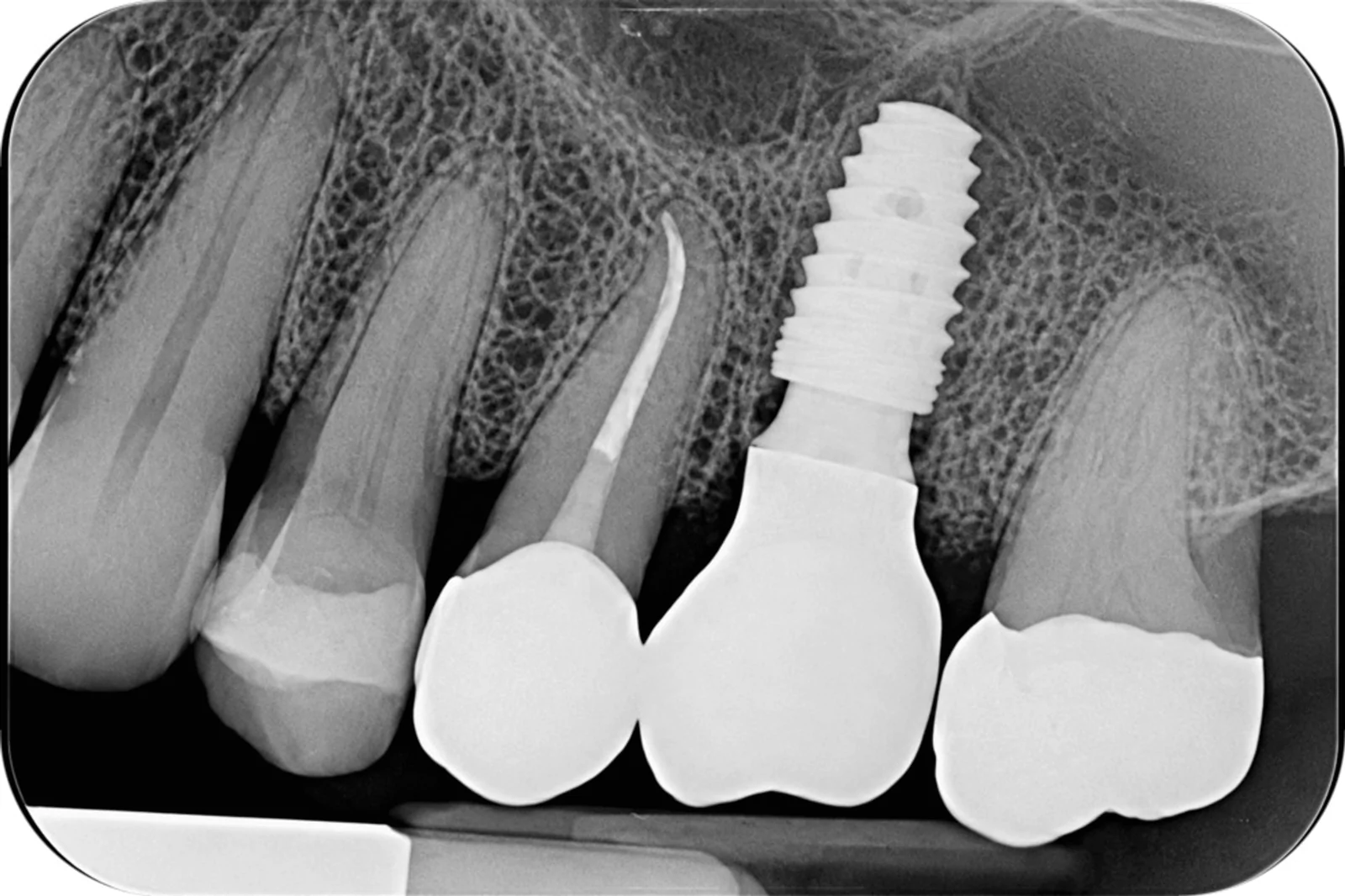
Radiograph at immediate implant placement and immediate provisionalization.

**Figure 6:**
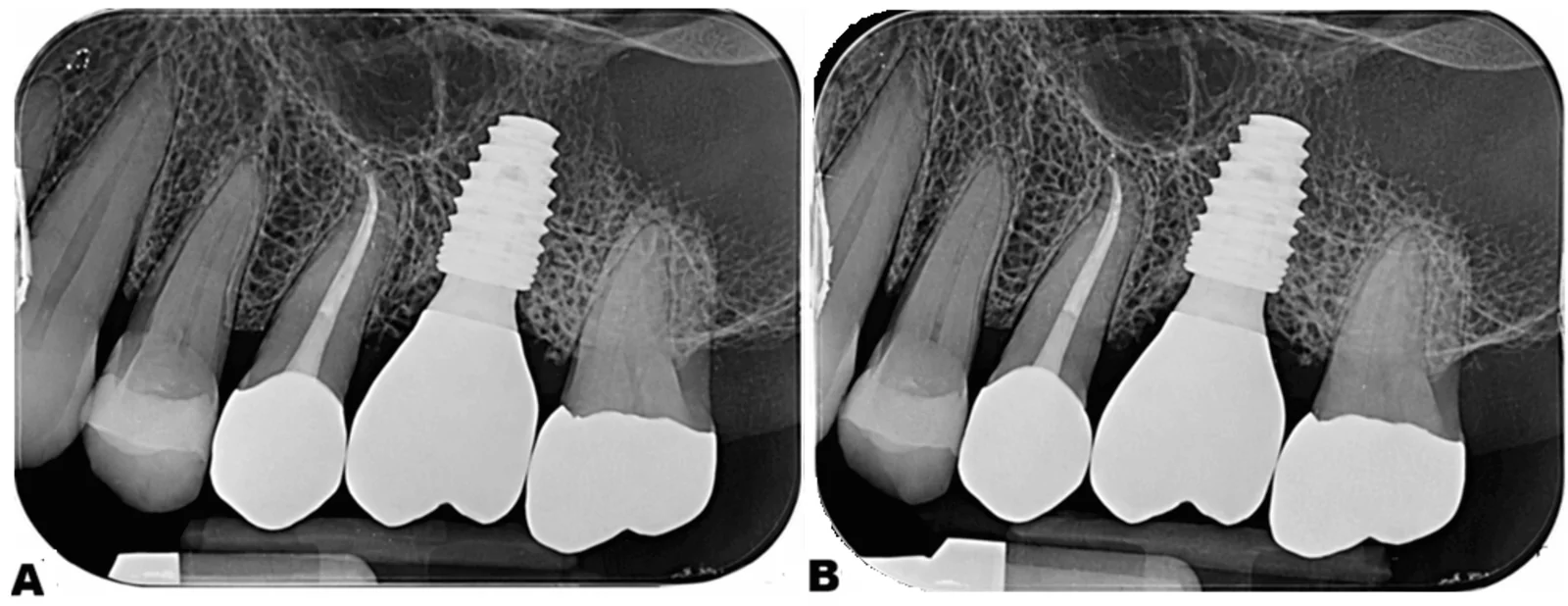
Maxillary periapical radiographic follow-up. (A) Radiograph at delivery of the definitive restoration. (B) Radiograph at 28 weeks after immediate implant placement and provisionalization, showing the crestal bone relative to the implant platform.

**Figure 7:**
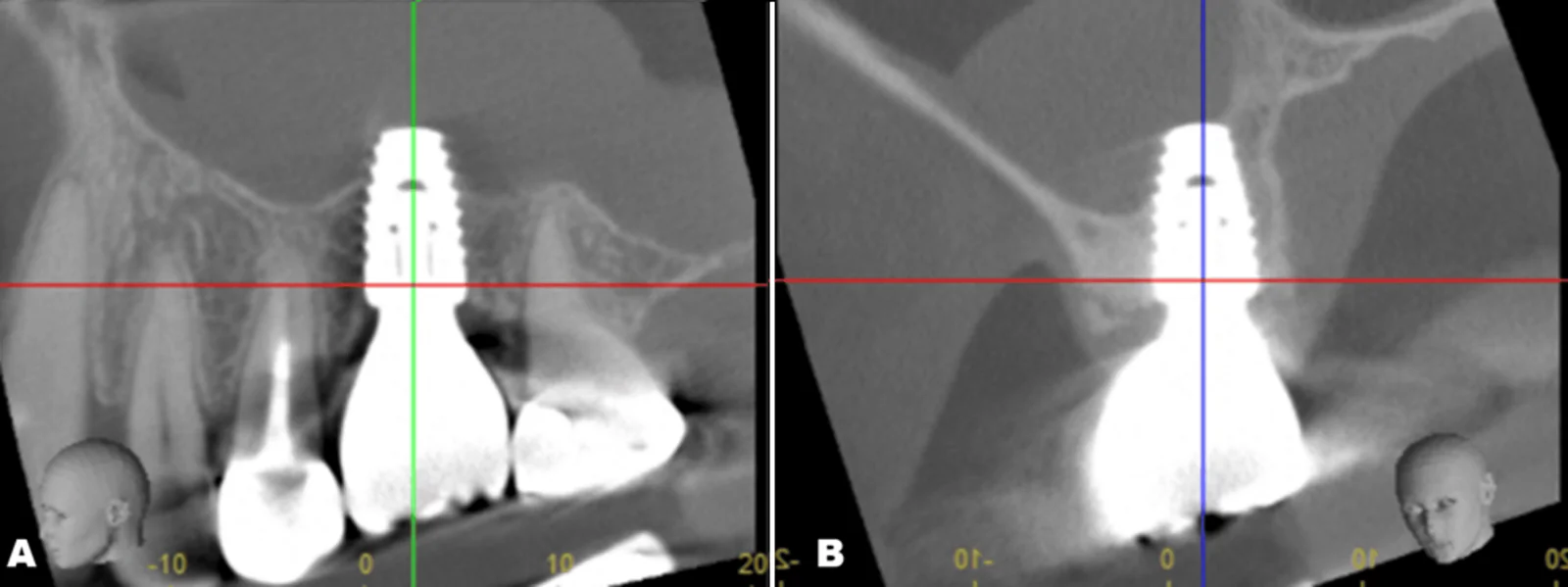
Maxillary CBCT assessment at 53 weeks. (A) Reconstructed view showing the implant and surrounding posterior maxillary anatomy. (B) Cross-sectional view showing the implant relative to the sinus and augmented region. CBCT: cone beam computed tomography.

**Figure 8:**
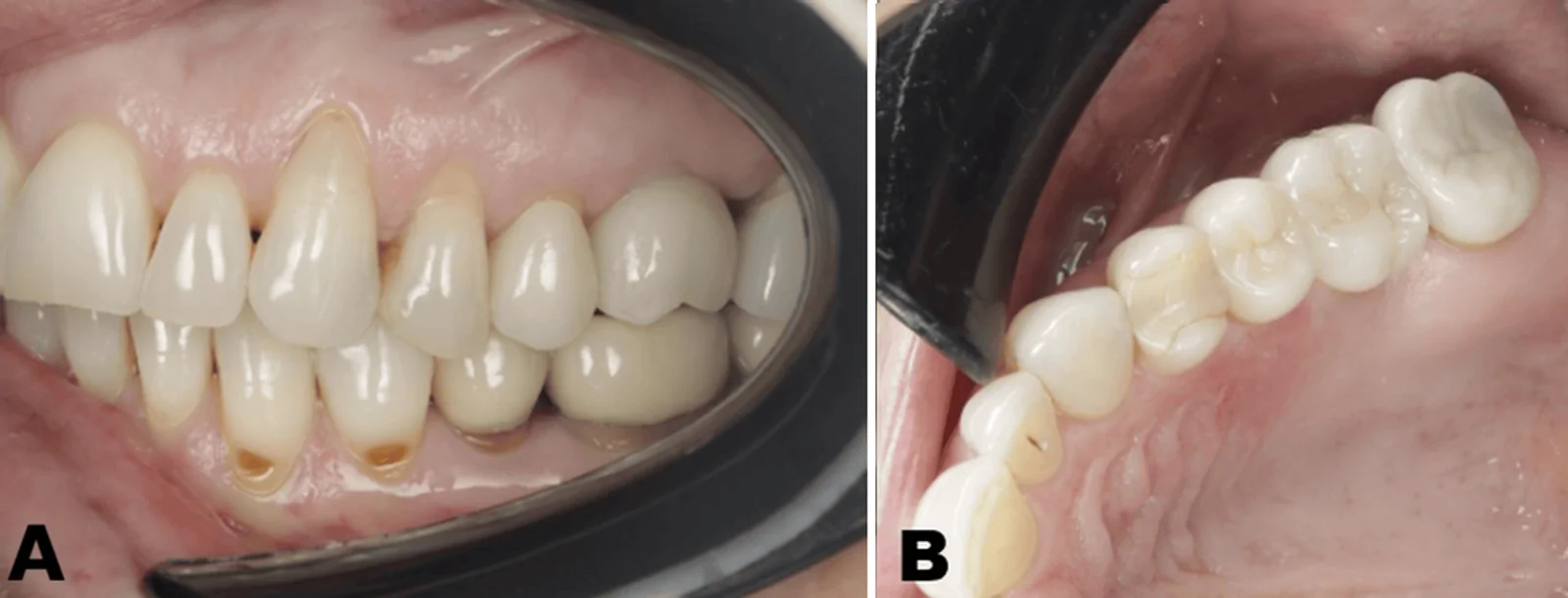
Maxillary clinical follow-up at 53 weeks. (A) Buccal view of the restored dentition and peri-implant gingiva. (B) Occlusal view of the maxillary arch and restoration. No visible peri-implant gingival inflammation is apparent in these views.

The treating author stated that follow-up CBCT was obtained to assess three-dimensional peri-implant bone levels. Standardized quantitative measurements were not supplied; the images were described as appearances, without inferring measured preservation or histologic regeneration. Photographs alone did not establish peri-implant health.

Illustrated case 2 mandibular first molar

A 58-year-old woman presented with recurrent caries and structural compromise at a previously endodontically treated mandibular first molar (Figure 9). The preoperative radiograph demonstrated periapical pathology associated with the failing restored tooth; the clinical history included previous apical surgery (Figure 10).

**Figure 9:**
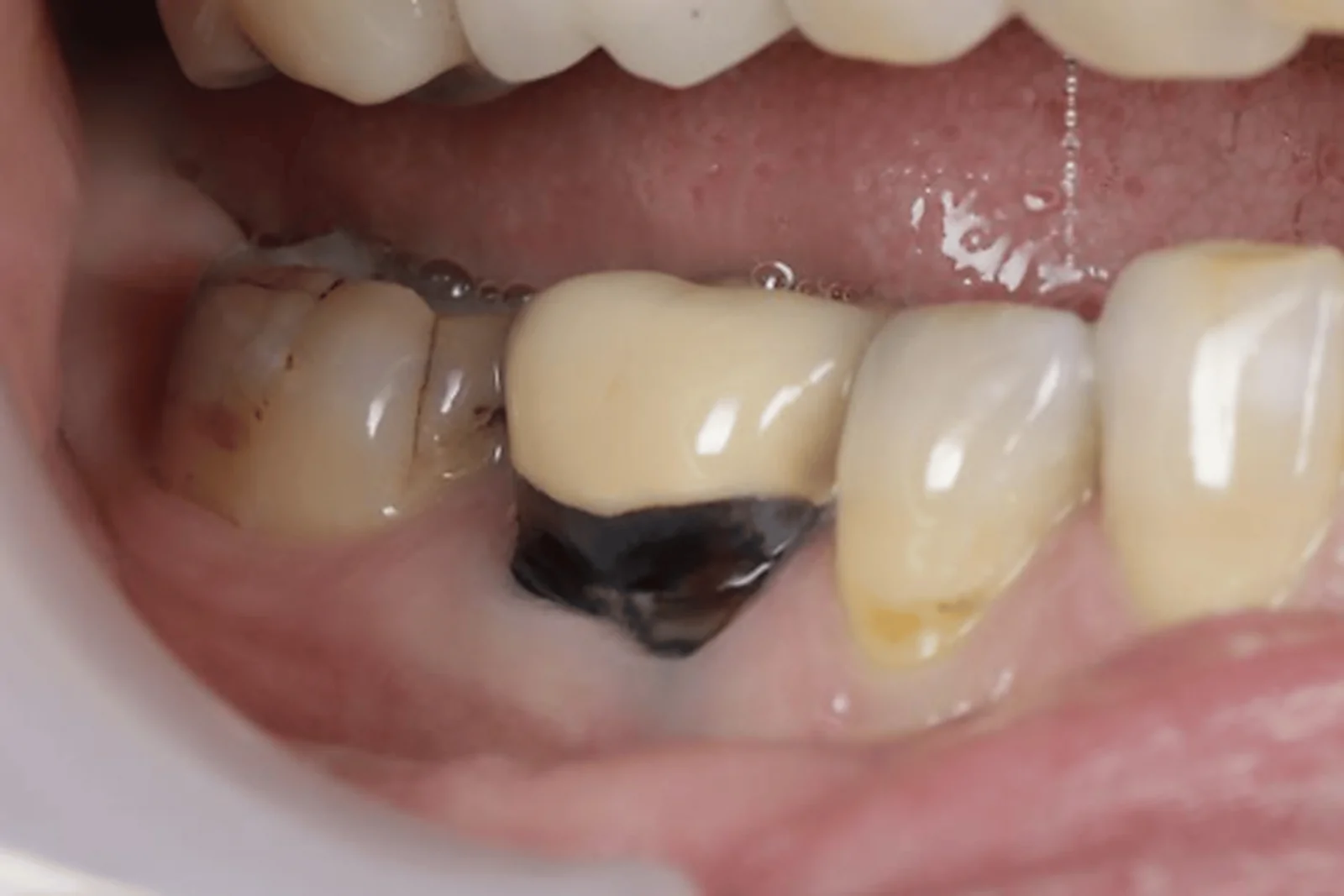
Clinical appearance of recurrent decay on the crown at the mandibular first molar.

**Figure 10:**
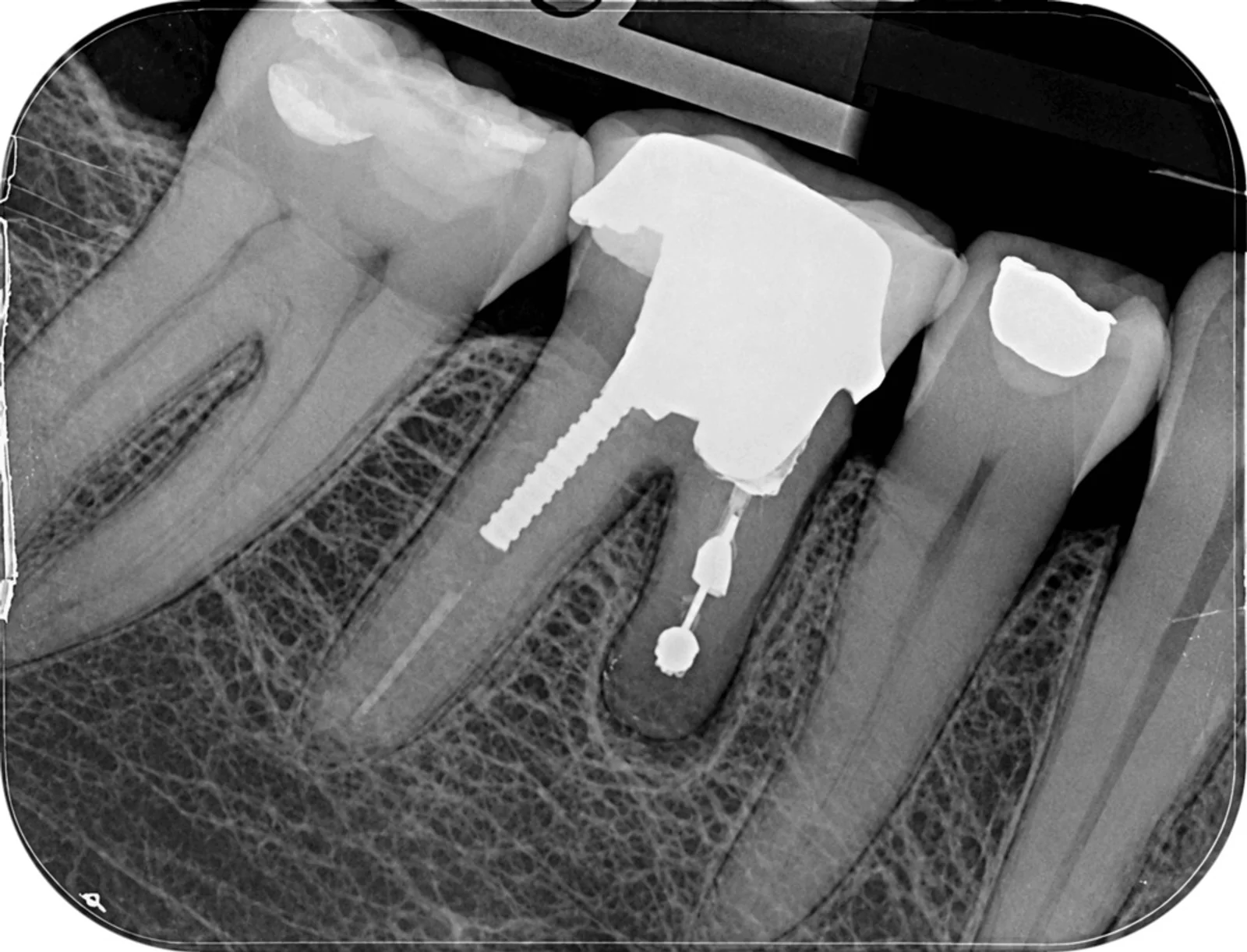
Radiograph of the previously endodontic and restored mandibular first molar with associated periapical infection related to multiple failed apicectomies.

The treating author identified this illustrated patient as mandibular case 3 in Table 2. That row recorded a CONELOG Progressive-Line implant measuring 5.0 × 11 mm with an insertion torque of 35 N·cm. The illustrated narrative instead described a BioHorizons implant measuring 5.2 × 10.5 mm. The implant identity and dimensions must be reconciled against the original record. CBCT-based virtual planning was used to establish a prosthetically appropriate implant trajectory while evaluating the relationship to the inferior alveolar canal (Figure 11A, B). After extraction and socket debridement, the implant was immediately placed in the planned position (Figure 12A).

**Figure 11:**
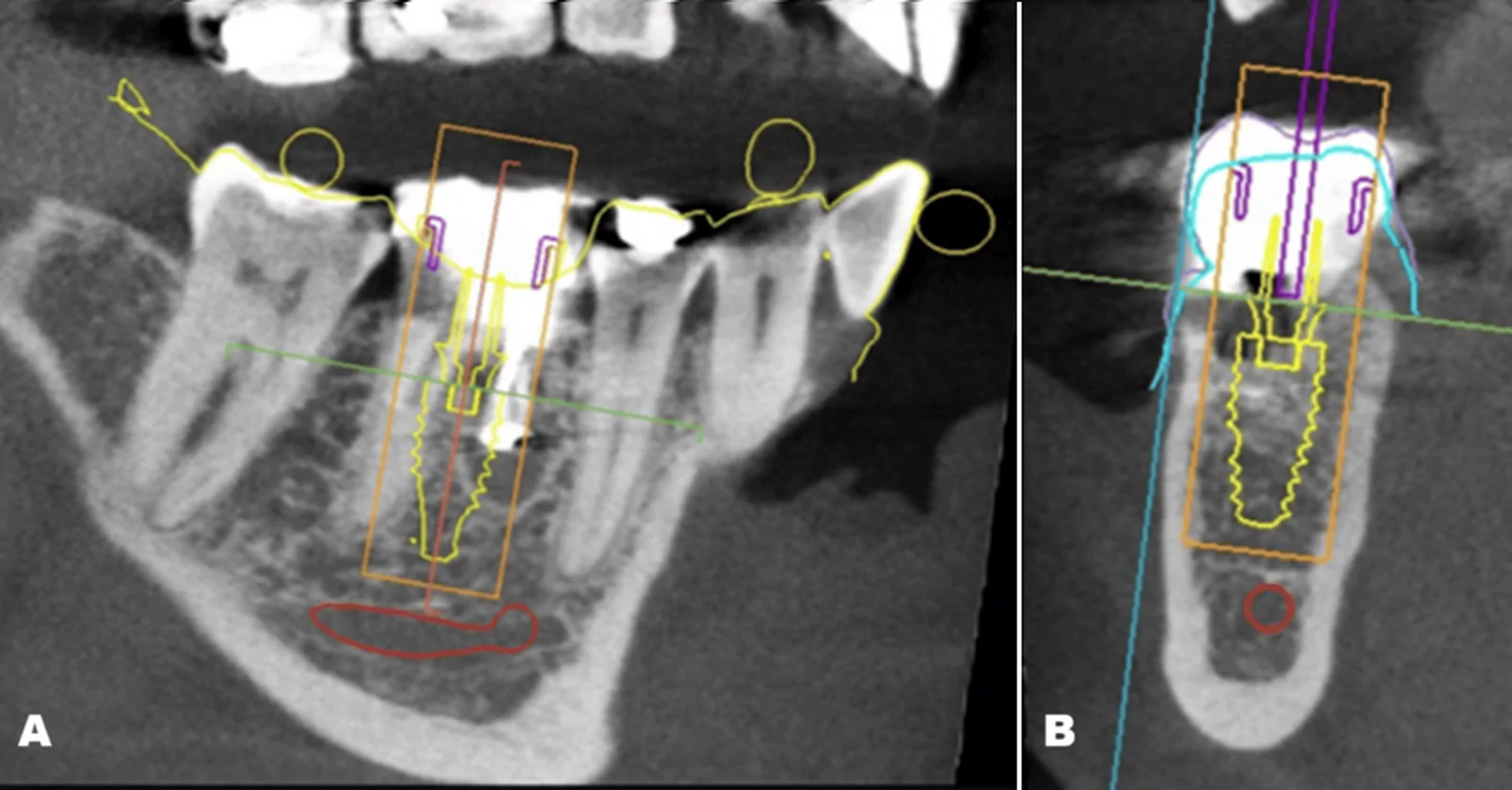
Mandibular virtual implant planning. (A) Reconstructed view showing the proposed implant position relative to the adjacent teeth and inferior alveolar canal. (B) Cross-sectional view of the planned implant trajectory and available bone above the canal.

**Figure 12:**
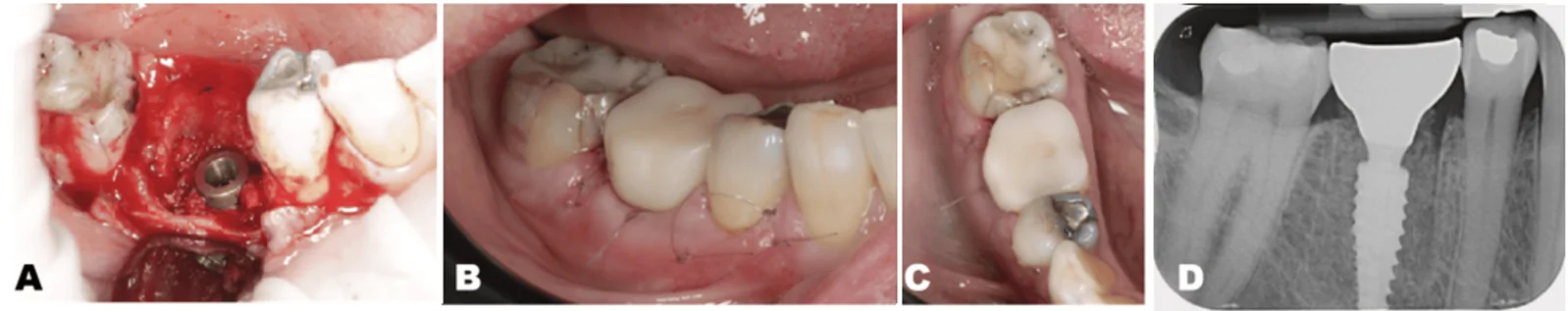
Mandibular immediate implant placement and provisionalization. (A) Occlusal view after extraction and immediate implant placement, before provisional connection. (B) Buccal view of the immediately connected provisional restoration. (C) Occlusal view of the provisional restoration and adjacent teeth. (D) Periapical radiograph at implant placement and immediate provisionalization.

The planned or achieved implant-to-canal distance was not supplied; an objective safety margin could not be reported. The osteotomy was prepared using guided surgery and surgical navigation. A BioHorizons Tapered Pro Conical implant measuring 5.2 × 10.5 mm (BioHorizons Implant Systems, Inc., Birmingham, AL) with a regular connection was placed. The residual implant-socket gap was grafted with autogenous bone, chopped L-PRF membrane, and MinerOss cortical and cancellous chips hydrated with PRF. A connective tissue graft harvested from the palate was added, and an anatomically contoured zirconia provisional restoration was immediately connected (Figure 12B, C). A placement radiograph documented the implant and provisional restoration (Figure 12D). During early healing, the soft tissue showed no visible inflammation and radiographs documented the crestal bone appearance (Figure 13A, B). The provisional was removed for an intraoral scan and then reinserted while the definitive screw-retained restoration was fabricated. Figure 14 documents the restorative appointment. After delivery of the definitive restoration, the clinical records described no visible gingival inflammation or gingival bleeding at follow-up (Figure 15A, B). A CBCT examination at two years documented the bone appearance relative to the implant platform (Figure 16A, B).

**Figure 13:**
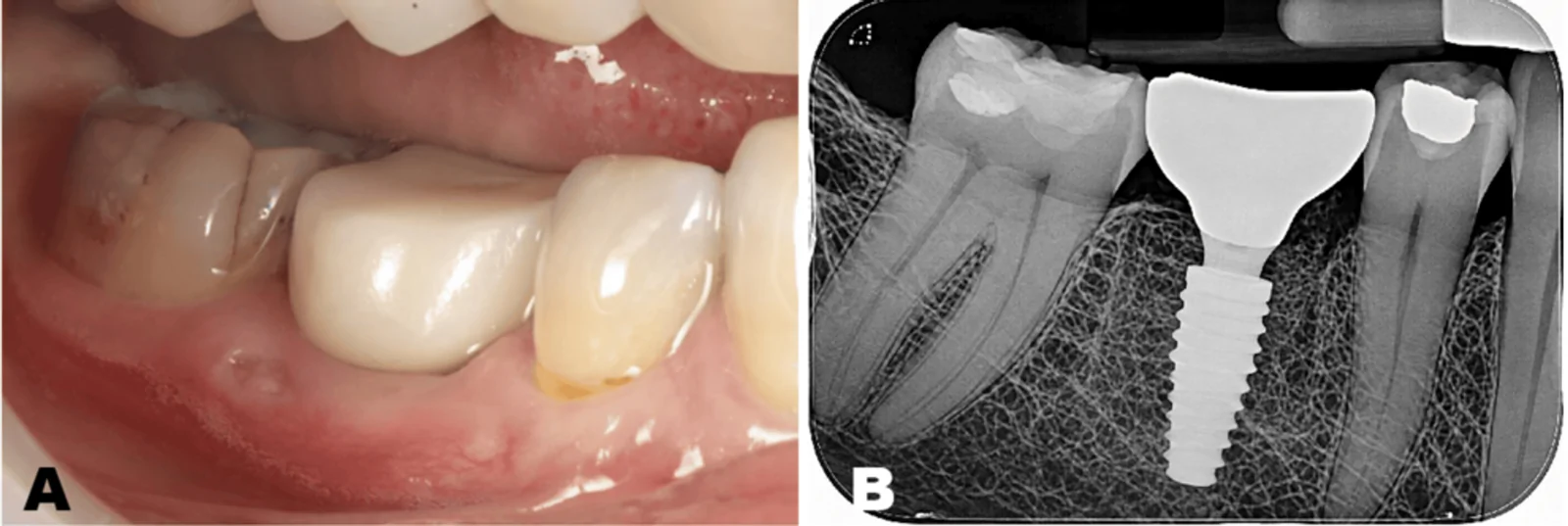
Mandibular review 12.5 weeks after immediate implant placement and provisionalization. (A) Buccal clinical view showing the peri-implant soft tissue around the provisional restoration. (B) Periapical radiograph showing the crestal bone relative to the implant platform.

**Figure 14:**
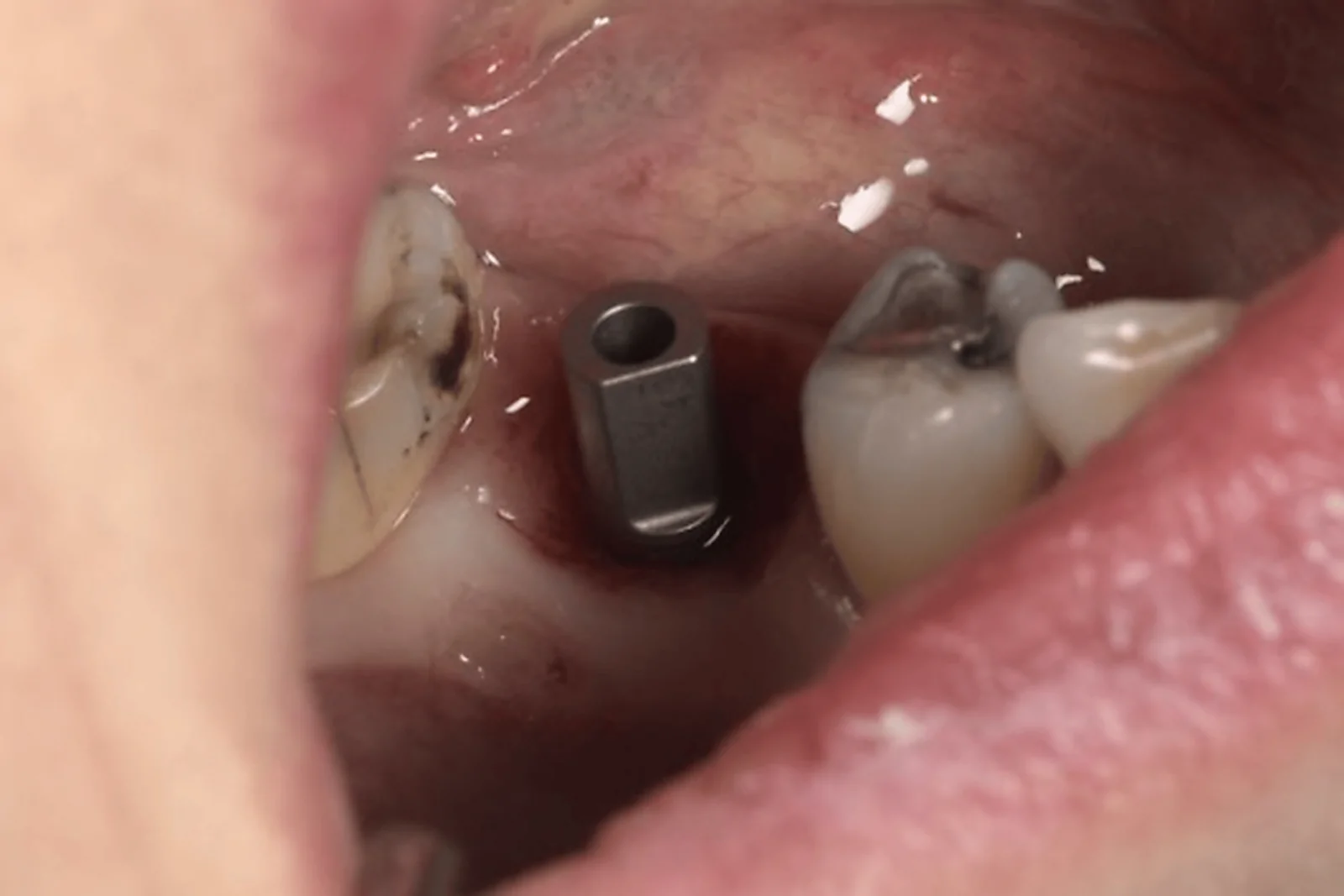
Clinical appearance after provisional removal and abutment placement at the restorative appointment.

**Figure 15:**
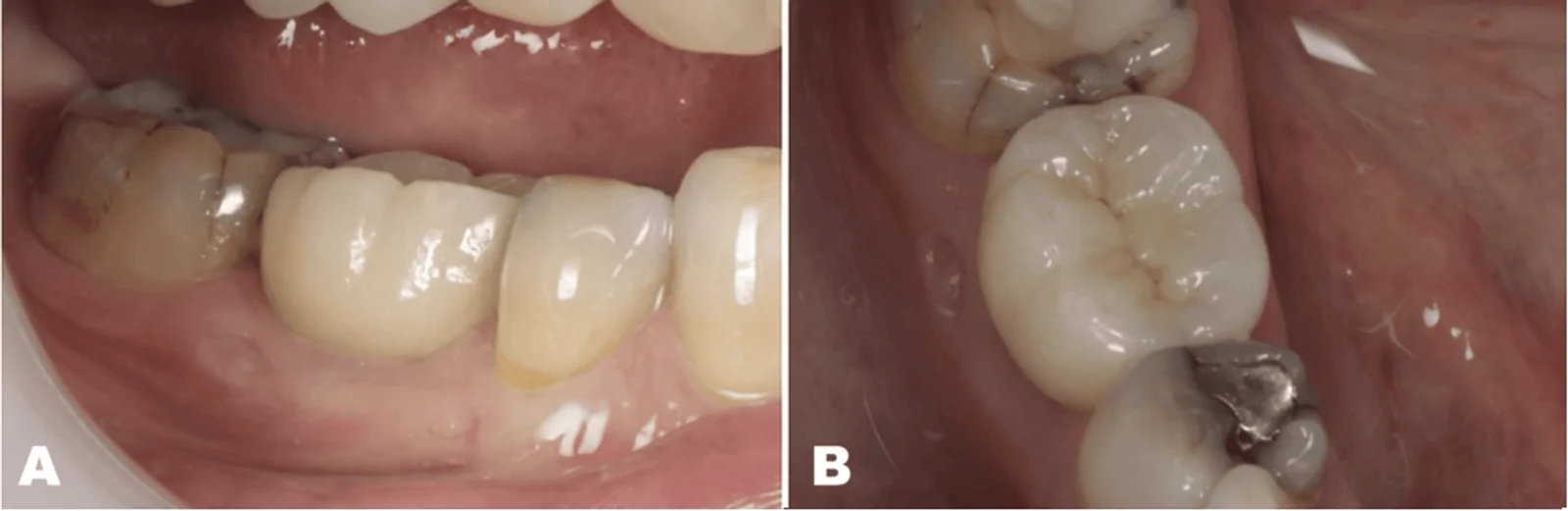
Mandibular definitive restoration at follow-up. (A) Buccal clinical view of the definitive restoration and surrounding peri-implant gingiva. (B) Occlusal view of the definitive restoration and adjacent teeth. No visible gingival inflammation is apparent in these views.

**Figure 16:**
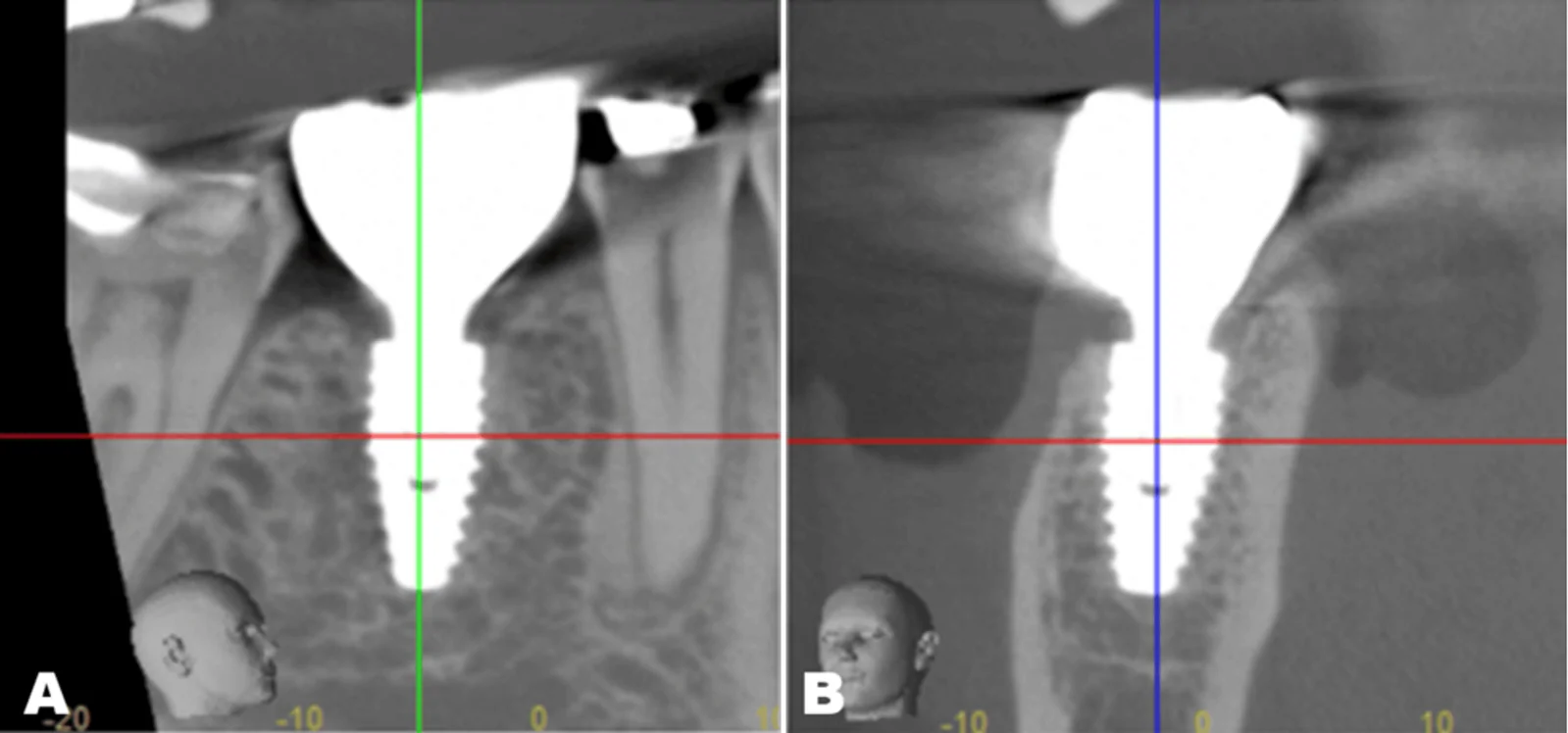
Mandibular CBCT evaluation two years after immediate implant placement and restoration. (A) Reconstructed view showing the restored implant and surrounding bone. (B) Cross-sectional view showing the peri-implant bone relative to the implant platform. Beam-hardening and related metal artifacts limit assessment of small peri-implant bone changes. CBCT: cone beam computed tomography.

The patient was linked to mandibular case 3, for which the recorded torque was 35 N·cm. The narrative implant system and dimensions remained discrepant from that row. A custom guide was used; any additional navigation, PRF preparation, specific graft indication, and scanning date require confirmation. The palatal designation referred to the donor site. Follow-up CBCT was obtained to assess three-dimensional peri-implant bone levels; metal artifacts and the absence of standardized measurements limited interpretation.

## Discussion

The series describes the application of a restoratively driven immediate protocol in private practice across both arches, including numerous sites with recorded infection and selective tissue augmentation. The lead author performed all procedures and managed or reviewed the available follow-up records using a postoperative schedule of two- and eight-week reviews and initiation of the restorative phase at 12 weeks. The two illustrated cases provide additional treatment detail and longitudinal imaging. Published reviews support immediate placement in selected infected sockets, although meticulous debridement, appropriate positioning, and adequate primary stability remain central considerations [6-9]. Published controlled studies and systematic reviews provided external clinical context; they did not validate this dataset or establish the effect of any individual component of its workflow.

Primary stability guided the timing of immediate provisionalization or custom healing abutment placement. In mandibular case 20, inadequate primary stability led to delayed custom healing abutment placement. The source record should not be interpreted as evidence of implant failure solely because the immediate restorative step was deferred. The maxillary sinus and inferior alveolar canal required different anatomic adaptations in the illustrated cases, emphasizing the need to identify available native bone and establish the restorative endpoint before surgery [3-5].

The provisional restoration is an important part of this technique. When properly contoured, it helps close the extraction socket, support the soft tissue, maintain proximal relationships, and develop the emergence profile for the definitive restoration. Finelle et al. described a digital CAD/CAM workflow for customized socket-sealing healing abutments in immediate molar sites [10]. The present protocol uses the same general tissue-support concept while providing an anatomically contoured immediate tooth replacement when primary stability permits.

Customized socket-sealing healing abutments and anatomically contoured provisional crowns were related tissue management approaches but differed in design, material, loading, and endpoints; they were not treated as equivalent interventions. Immediate provisionalization should not be confused with immediate functional loading. In this protocol, the provisional restoration was intended to be kept out of functional occlusion during early healing. This is particularly important in the posterior region, where occlusal forces are greater and an implant can be subjected to unfavorable loading before osseointegration has occurred [3,10].

The published molar data are encouraging, but patient and site selection remain important. Ragucci et al. reported high short-term survival and success in their systematic review and also identified factors related to gap grafting, loading protocol, and the buccal bone [3]. Fugazzotto reported favorable results in large maxillary and mandibular molar groups treated at the time of extraction, with regenerative treatment added when needed [4,5]. Taken together, these reports support immediate molar placement in properly selected sites, but they do not suggest that every molar extraction socket should be treated immediately.

Another reason to consider immediate treatment is what happens to the ridge after extraction. Human studies have demonstrated substantial horizontal dimensional change during the first months after a tooth is removed [1]. With a staged approach, some of the anatomy present on the day of extraction may later need to be recreated with augmentation. Immediate placement does not prevent normal post-extraction remodeling, but it allows implant positioning, grafting, and tissue support to begin while the original socket anatomy and tooth position are still available to guide treatment.

Hard- and soft-tissue augmentation was selected according to the defect and tissue requirements. The maxillary case included crestal sinus augmentation, socket grafting, and a connective tissue graft from the tuberosity. The mandibular case combined socket grafting with a palatal connective tissue graft. These adjuncts were used for management of the residual defect; they should not be used to compensate for an unstable or incorrectly positioned implant [3-5,11,12]. Variation in implant system, guidance, graft materials, sinus elevation, platelet preparations, soft-tissue augmentation, and restoration type prevented attribution of the observations to immediate placement or immediate provisionalization alone.

The available images document favorable clinical and radiographic findings at the reported evaluations, but treatment timing and outcomes cannot be compared with a staged protocol in this report. Evidence on the timing of implant placement remains relevant when discussing alternatives with patients [11]. A recent systematic review of damaged extraction sockets also emphasizes the importance of site selection when considering immediate placement [12].

Placement timing and loading timing should be assessed together. Gallucci et al. evaluated these as combined protocols and found that the loading approach influences interpretation of immediate-placement outcomes [13]. Accordingly, the present series describes planned nonfunctional provisionalization; its insertion torque records cannot establish the safety of immediate functional loading or predict long-term survival. Insertion torque alone also cannot establish osseointegration or the safety and long-term outcome of nonfunctional provisionalization.

Limitations

This retrospective series provides clinical experience from private practice with a consistent treating and follow-up clinician. The cases varied in arch, implant system, provisional material, and augmentation requirements. Although the early postoperative schedule was consistent and the authors clarified the reported complications, complete final observation periods, standardized success or survival definitions, quantitative bone-level measurements, and validated patient-reported outcomes were not supplied. The treating author confirmed that 46 patients, one site per patient, were treated over the preceding five years. Consecutive inclusion remained unconfirmed. One infection status and some soft-tissue graft fields were missing. Narrative and tabular discrepancies in the illustrated maxillary case require reconciliation with the clinical records. These features limit estimates of long-term outcomes and comparative effectiveness while preserving the value of the series as a description of clinical application.

The treating author confirmed 46 patients with one listed site per patient, a five-year treatment period, no loss to follow-up, and arch-level follow-up as described above. Consecutive inclusion remained unspecified. The lack of standardized probing, longitudinal quantitative bone measurements, and per-site observation dates limited interpretation. The linked illustrated cases contained implant or torque discrepancies, and maxillary case 15 required reconciliation with the stated stability threshold.

## Conclusions

This retrospective private practice series documents 46 molar treatment sites in 46 patients treated over the preceding five years managed with planned immediate implant placement and immediate nonfunctional provisionalization or custom healing abutment placement. All cases were treated, verified, and followed by the lead author. Reported postoperative complications included pain, a loose custom healing abutment corrected by tightening, and slight peri-implant recession. In one mandibular site, inadequate primary stability prompted delayed custom healing abutment placement. The illustrated cases demonstrate the application in both arches and favorable findings at their reported follow-up evaluations.

The findings describe a clinical workflow incorporating site selection, digital planning, selective augmentation, and scheduled postoperative review. Immediate restorative steps should be adapted to the primary stability obtained at placement. Complete outcome records and final observation periods are needed to establish implant survival, time-dependent complication rates, and comparative benefits. The current dataset supports the description of clinical application only and does not establish comparative effectiveness, safety, or long-term predictability.
